# Clinical Evaluation of Reasons for Replacement of Amalgam Restorations in Patients Referring to a Dental School in Iran

**DOI:** 10.5681/joddd.2010.015

**Published:** 2010-06-24

**Authors:** Firoz Pouralibaba, Mohammad Joulaei, Atabak Kashefimehr, Farzaneh Pakdel, Zahra Jamali, Ali Esmaeili

**Affiliations:** ^1^ Assistant Professor, Department of Oral Medicine, Faculty of Dentistry, Tabriz University of Medical Sciences, Tabriz, Iran; ^2^ Assistant Professor, Department of Operative Dentistry, Faculty of Dentistry, Zanjan University of Medical Sciences, Zanjan, Iran; ^3^ Assistant Professor, Department of Periodontics, Faculty of Dentistry, Tabriz University of Medical Sciences, Tabriz, Iran; ^4^ Postgraduate Student, Department of Oral Medicine, Faculty of Dentistry, Tabriz University of Medical Sciences, Tabriz, Iran; ^5^ Assistant Professor, Department of Prosthodontics, Faculty of Dentistry, Tabriz University of Medical Sciences, Tabriz, Iran

**Keywords:** Amalgam failures, cusp fracture, restoration replacement

## Abstract

**Background and aims:**

The present study evaluated the most common reasons for replacing amalgam restorations in a university clinic.

**Materials and methods:**

A total of 217 restorations which needed to be replaced were clinically and radiographically evaluated in a period of 4 months. The frequencies of reasons for replacing amalgam restorations were calculated: The assessed items included recurrent caries, tooth structure fracture (functional or non-functional cusps), amalgam bulk fracture, amalgam marginal fracture, proximal overhangs, and esthetics. Data were analyzed using Fischer’s exact test.

**Results:**

Both in vital teeth and teeth which had undergone root canal therapy, the most common reason for amalgam replacement was cusp fracture, with the fracture of non-functional cusps being statistically significant. Recurrent caries was the second most common reason for amalgam replacement. In Class I restorations, the most common reasons were recurrent caries and esthetics, with no statistical significance. The most frequent problem in Class II restorations was fracture of non-functional cusps, with a statistical significance in three-surface restorations.

**Conclusion:**

According to the results, failing to reduce undermined cusps and neglectful caries removal are the reasons for majority of amalgam restoration replacements. These issues should be emphasized in the curriculum for dental students and continuing education courses.

## Introduction

Amalgam restorations are one of the most common dental restorations. Despite all the controversies regarding adverse effects of mercury on health, amalgam has retained its position as one of the most commonly used restorative materials and there is still demand for such restorations. The popularity of amalgam might be attributed to its good mechanical properties, ease of application and the one-appointment treatment.^1^ Amalgam has low technical sensitivity and it is believed that metallic ions released by amalgam have anti-cariogenic activity.^2^ However, amalgam cannot strengthen tooth structure, and is unaesthetic. In addition, cavity preparation for amalgam is difficult and proper insulation is necessary.

It has been demonstrated that a large number of patients referring to operative dentistry departments have faulty restorations, including amalgam restorations, needing replacement. A restoration which cannot restore function, esthetic and phonetic should be replaced.^1^ Failure of dental restorations is of major concern in the dental practice. Survival and failure rates may be used as measures of clinical performance. The reason for failure of restorations is also of importance, because it points to a specific weakness of the restoration-tooth system.

Since prevention has priority over treatment, a proper knowledge about the etiologic factors involved in failures, will lead to successful amalgam restorations. Although numerous in vitro studies have been carried out on the etiologic factors involved in the failure of amalgam restorations, all evaluating such restorations from different aspects, there is need for clinical studies. The present clinical investigation evaluated the reasons for replacing failed amalgam restorations in patients referring to a university clinic.

## Materials and methods

This cross-sectional study encompassed 100 patients (217 restored teeth), selected continuously from patients referring to the Department of Operative Dentistry, Dental Faculty of Zahedan University of Medical Sciences, Zahedan, Iran, during a four-month period. All subjects were carefully examined and their dental records were reviewed. The inclusion criteria included faulty amalgam restorations, placed at least 18 months before the time of examination. Patients had a chief complaint of either replacing their tooth restorations or other complaints, though they had faulty restorations needing replacement. In the latter case, the subjects were informed, and then, included in the study. Each subject had 1-4 faulty restorations. The reasons to replace restorations included: 1. recurrent caries; 2. fracture of tooth structure (functional or non-functional cups); 3. amalgam bulk fracture; 4. fracture of amalgam margin; 5. proximal overhangs; and 6. esthetic reasons (The subject was not satisfied with the esthetic appearance of amalgam restorations). In cases in which the cusp/cusps had fractured due to recurrent caries, “recurrent caries” was reported as the reason for restoration replacement.

All evaluations were done by one operator. Dental mirrors and explorers were used for clinical examinations after drying the teeth with air spray. Dental floss was used to evaluate proximal restorations. Also, periapical and bite-wing radiographs were used for radiographic evaluation of restorations/teeth. Subjects were asked when the restorations had been placed. Endodontic treatment of the teeth was also recorded.

Frequencies of reasons for the replacement of restorations were calculated. Also, the frequencies for endodontically treated/untreated teeth as well as each restoration type were calculated separately. Data were analyzed using Fischer’s exact test. A P-value of <0.05 was considered significant.

## Results

Of all 217 teeth evaluated, 131 (60.36%) teeth were vital and 86 (39.63%) were endodontically treated (Table 1). The evaluated teeth revealed 35 (16.12%) one-surface, 79 (36.41%) two-surface, and 103 (47.47%) three-surface restorations, respectively (Table 2).

**Table 1:** Frequencies of reasons for replacing amalgam restorations in all teeth evaluated according to tooth vitality

	All teeth (n = 217)		Vital teeth (n = 131)		Endodontically-treated teeth (n = 86)	
Reason for replacement of restoration	Frequency	P value^*^	Frequency	P value^*^	Frequency	P value^*^
Recurrent caries	40 (18.43%)	0.243	26 (19.85%)	0.0029	14 (16.27%)	0.202
Functional cusp fracture	46 (21.2%)	0.065	26 (19.85%)	0.0029	20 (23.25%)	0.112
Non-functional cusp fracture	64(29.49%)	0.003	28 (21.37%)	0.0018	36 (41.86%)	0.0012
Amalgam bulk fracture	22 (10.14%)	0.365	20 (15.26%)	0.156	2 (2.32%)	0.379
Amalgam marginal fracture	2 (0.33%)	0.633	2 (1.53%)	0.554	0	0
Proximal overhang	25 (11.52%)	0.302	11 (8.39%)	0.490	14 (16.27%)	0.202
Esthetics	18 (8.29%)	0.464	18 (13.75%)	0.413	0	0

^* ^P value from Fisher exact test

**Table 2:** Frequencies of reasons for replacing amalgam restorations according to the type of restorations evaluated

		Type of restoration	
Reason for replacement of restoration	1-surface (n=35)	2-surface (n=79)	3-surface (n=103)
Recurrent caries	11 (31.43%)	12 (15.18%)	17 (16.5%)
Functional cusp fracture	1 (2.85%)	17 (21.52%)	28 (27.18)
Non-functional cusp fracture	3 (8.57%)	21 (26.58%)	40 (38.84%)
Amalgam bulk fracture	7 (20%)	9 (11.4%)	6 (5.89%)
Amalgam marginal fracture	2 (5.72%)	0	0
Proximal overhang	0	14 (17.72%)	11 (10.68%)
Esthetics	11 (31.43)	6 (7.6%)	1 (0.97%)

The three most prevalent reasons for restoration replacement in all the teeth were fracture of non-functional cusps, fracture of functional cusps, and recurrent caries, respectively. Fracture of non-functional cusp was found to be statistically significant among all reasons evaluated. “Amalgam marginal fracture” and “esthetics” were not observed in endodontically treated teeth.

In one-surface restorations, the most common reasons for replacing of restorations were recurrent caries and esthetics, which were not statistically significant. The most prevalent failure in two and three-surface (MO, DO, and MOD) restorations, was the fracture of non-functional cusps, which was statistically significant for MOD restorations. Marginal fracture was not observed in MO, DO, and MOD amalgam restorations.

## Discussion

The minimum amalgam restoration longevity was considered to be 18 months in the present study, since this is the period amalgam, as an appropriate restorative material, should withstand 1.5 million force cycles in the oral cavity. Therefore, only those restorations were included in the study which had been subjected to this minimum period of withstanding cycles of mastication forces.^3^

According to the results of the present study, the most common reason for restoration failure in both endodontically treated and vital teeth was cusp fracture, especially fracture of non-functional cusps, which has a linear relationship with the size of the cavity and restoration. Therefore, undermined cusps should be reduced and capped with restorative material to minimize the odds of fracture. Non-functional cusps undergo the most severe fractures.^1^ In the present study, most of cusp fractures were so extensive that full coverage restorations were necessary to restore the tooth.

Recurrent caries was the second most common reason for replacing restorations. Recurrent caries can result from inadequate removal of caries during restorative procedures or might be new lesions under restorations. Therefore, meticulous removal of carious lesions during restorative procedures should be of utmost importance. Routine caries removal procedures can be supplemented with adjunctive methods, including caries detector solutions,^4^ and polymer burs.^1^ Another approach, especially in high-risk patients, is regular follow-up visits. It should be emphasized that follow-up visits are an integral part of the treatment procedure and have a great role in the treatment success.^2^

In a recent study comparing two amalgam materials based on United States Public Health Service (USPHS) standards (recurrent caries, marginal adaptation, anatomic form, surface appearance and surface shine), recurrent caries was more prevalent than other clinical criteria after one year.^5^ A seven-year evaluation of the survival rate of the amalgam and composite restorations also revealed that reasons of failure were either secondary (recurrent) caries or restoration fracture, with secondary caries being the main reason for failure in both of amalgam and composite restorations.^6^ In our study, the main reason for failure was cusp fracture, probably because majority of subjects had large restorations.

A15-year follow-up on 1213 Class II amalgam restorations showed that approximately 17.6% of the cases had undergone restoration replacement procedures.^7^ Factors influencing the replacement rates were found to be gender, type of restoration (MO or DO vs. MOD) and operator.^7^ In the present study, gender was not considered as a factor; however, the type of restoration and the operator were found to be important factors influencing durability of restorations.

Kolker et al^8^ concluded from an evaluation of 5-10-year-old extensive amalgam restorations that such teeth should undergo a full-coverage procedure after 5 years to prevent restoration failure. Another study on 300 extensive amalgam restorations in a 4-year period revealed a 10% failure rate.^9^ The results of the study showed that with a proper restorative technique and coverage of undermined cusps, good clinical results can be obtained and there is no need for cast restorations at least in the first 4 years to strength the remaining tooth structure.^9^ These findings emphasize the importance of reduction of weak tooth structures and their capping with restorative materials.

Plasmans et al^10^ evaluated the 100-month success rate of amalgam restorations and concluded that success of extensive amalgam restorations alone or as a foundation for cast procedures shows no statistically significant differences. In addition, failure rate of amalgam restorations in older patients is higher than that in youngers.^10^

An important finding in the present study was the low frequency of cusp-coverage restorations, which might be the reason for a high rate of cusp fractures. A recent similar study in Iran on two-surface amalgam restorations showed that the most common reasons for restoration failures were, in descending order, proximal overhangs, recurrent caries and food impaction.^11^ However, in the present study, the most common failure reasons, in descending order, were cusp fractures, recurrent caries and proximal overhangs. The higher frequency of cusp fractures in our study compared to the latter study might be due to our inclusion of endodontically-treated teeth and three-surface restorations.

Evaluating the reasons for replacing amalgam, composite resin and glass-ionomer restorations, Mjör^12^ reported recurrent caries as the most common reason for restoration replacement. In another study on 3455 restorations rendered by 48 dental practitioners, 65% of the restorations had been carried out to replace an existing restoration.^13^ Recurrent caries was the most common reason followed by tooth or restoration fracture. The mean age of the restorations was 15 years.^13^ Proximal overhang which can lead to periodontal problems and recurrent caries, might be avoided by the use of wedges and contouring of matrix bands. The rate of amalgam margin fractures has decreased with the introduction of high-copper amalgams,^3^ which is confirmed by the results of the present and previous studies. However, this should not overshadow the importance of proper carving, burnishing and polishing of amalgam restorations.

Another reason for replacing restorations in the present study was amalgam bulk fracture, which might be attributed to improper cavity design or recurrent caries resulting in undermining of the amalgam restoration. Amalgam cannot withstand tensional forces well; therefore, cavities should be prepared in a manner in which the material will have sufficient thickness and will not be subjected to tensional forces.^3^

Finally, some subjects were not satisfied with the esthetic appearance of amalgam restorations and had decided to have them replaced. Such subjects did not have extensive restorations and seemed to have satisfactory oral health and hygiene status.

The findings of the present study emphasize the importance of proper education of dental practitioners and dental students in preventing restoration failures. Subjects like cusp coverage and importance of follow-up visits should be emphasized in the curriculum for dental students and continuing education courses for dental practitioners.
